# Navigating the ALS Genetic Labyrinth: The Role of MAPT Haplotypes

**DOI:** 10.3390/genes14112023

**Published:** 2023-10-30

**Authors:** Ivan Tourtourikov, Kristiyan Dabchev, Tihomir Todorov, Teodor Angelov, Teodora Chamova, Ivailo Tournev, Tanya Kadiyska, Vanyo Mitev, Albena Todorova

**Affiliations:** 1 Department of Medical Chemistry and Biochemistry, Medical University of Sofia, 1431 Sofia, Bulgaria; 2 Genetic Medico Diagnostic Laboratory Genica, 1612 Sofia, Bulgaria; 3 Faculty of Biology, Sofia University St. Kliment Ohridski, 1504 Sofia, Bulgaria; 4 Department of Neurology, Faculty of Medicine, Medical University of Sofia, 1431 Sofia, Bulgaria; 5 Department of Neurology, Clinic of Nervous Diseases, Medical University of Sofia, UMBAL Aleksandrovska, 1431 Sofia, Bulgaria; 6 Department of Cognitive Science and Psychology, New Bulgarian University, 1618 Sofia, Bulgaria; 7 Department of Physiology and Pathophysiology, Medical University of Sofia, 1431 Sofia, Bulgaria

**Keywords:** MAPT, ALS, H1 haplotype, neurodegenerative disorders

## Abstract

Amyotrophic lateral sclerosis (ALS) is a neurodegenerative disease characterized by wide clinical and biological heterogeneity, with a large proportion of ALS patients also exhibiting frontotemporal dementia (FTD) spectrum symptoms. This project aimed to characterize risk subtypes of the H1 haplotype within the *MAPT* (microtubule-associated protein tau) gene, according to their possible effect as a risk factor and as a modifying factor in relation to the age of disease onset. One hundred patients from Bulgaria with sporadic ALS were genotyped for the variants rs1467967, rs242557, rs1800547, rs3785883, rs2471738, and rs7521. Haploview 4.2 and SHEsisPlus were used to reconstruct haplotype frequencies using genotyping data from the 1000 Genomes project as controls. Genotype–phenotype correlation was investigated in the context of age of disease onset and risk of disease development. While the individual variants of the subtypes do not influence the age of onset of the disease, a correlation was found between the specific haplotype GGAGCA (H1b) and the risk of developing sALS, with results showing that individuals harboring this haplotype have a nearly two-fold increased risk of developing sALS compared to other H1 subtypes. The results from this study suggest that fine transcriptional regulation at the *MAPT* locus can influence the risk of ALS.

## 1. Introduction

Alterations in the *MAPT* (microtubule-associated protein tau) gene (OMIM#157140) are the cause of several neurodegenerative diseases such as progressive supranuclear palsy (SP), corticobasal degeneration (CD), frontotemporal dementia (FTD), Parkinson’s disease (PD), and late-onset Alzheimer’s disease (LOAD) [1]. The tau protein’s abnormal accumulation in the brain, leading to the formation of neurofibrillary tangles, is a hallmark of the above neurodegenerative diseases, also known as the main group of tauopathies [2], as well as others, such as amyotrophic lateral sclerosis (ALS) with or without FTD [3,4]. 

**Figure 1:**
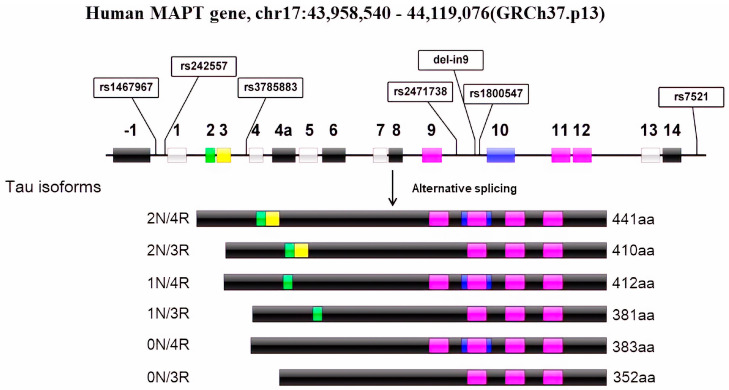
Tau protein gene structure, showing the tagging SNPs used to discriminate *MAPT* subhaplotypes and the different isoforms expressed in the human brain.

The first pathogenic/likely pathogenic variants in the gene were discovered in 1998 and were associated with a dominant form of frontotemporal dementia-17 (FTDP-17), and more than 50 pathogenic variants are now known to science, located mainly in exons 9–12 [5,6]. Such variants can affect the protein product of *MAPT*, both by altering microtubule-binding properties and at the RNA level, shifting splicing toward overproduction of specific tau isoforms [7]. In the adult human brain, tau has six different isoforms, ranging in length from 352 to 441 amino acids (Figure 1). They are characterized by the variable numbers of microtubule-binding domains, with three of them having three repeats of the respective domains (3R-tau) and three isoforms having four repeats (4R-tau) in the C-terminal half [8,9]. In a healthy person, these isoforms are expressed in a balanced manner, while in the various manifestations of tauopathies, this balance is shifted towards a specific profile of the isoforms and, accordingly, of the pathological tau deposits formed [10]. In addition to direct pathological changes in the gene, previous studies on patients from the listed group of neurodegenerative diseases worldwide demonstrate the presence of a risk haplotype defined by a deletion of 238 nucleotide bases in the intron between exons 9 and 10 (del-in9, Figure 1), as well as the presence of 20 subtypes characterized by variants in the intronic regions of *MAPT* [11]. The main haplotypes, H1 and H2, are defined by a 900 kb inversion, creating a block of linkage disequilibrium that suppresses recombination between the two haplotypes [12]. The variants used throughout the literature are rs1467967, rs242557, rs1800547, rs3785883, rs2471738, and rs7521, with rs1800547 used as a tagging SNP for the H1/H2 major type. Data suggest that these haplotypes contribute to increased disease risk through a modifying effect on the expression of the gene itself and, respectively, of its isoforms, contributing to heterogeneity in the clinical presentation of tau-associated neurodegenerative diseases [13]. These subtypes have been reported in the literature with specific associations, such as the H1c type for PSP and PD and H2 for AD [14,15].

Some studies have also reported a weak association between the H1 haplotype and AD [16]. Genome-wide association studies have confirmed the association between common variations in the α-synuclein gene (*SNCA*) and the *MAPT* H1 haplotype with sporadic PD [17]. Functionally, these associations are partly due to changes in tau expression and splicing, potentially affecting the selective deposition of 4R tau isoforms in diseases such as progressive supranuclear palsy and corticobasal degeneration [18]. Interestingly, these haplotypes have also been found to be associated with increased blood glucose levels [19] and a role of the *MAPT* locus on the 17q chromosome has been implied in food addiction [20]. While these associations highlight the diverse roles of the *MAPT* gene, its implications in other neurodegenerative diseases, particularly ALS, are also evident and merit further investigation [21]. ALS is a neurodegenerative disease affecting almost exclusively motor neurons. It is characterized by clinical and biological heterogeneity due to the wide spectrum of behavioral and motor symptoms that can manifest throughout the disease [22]. According to the literature, up to 85% of cases are sporadic (sALS) [23], and of this group, 50% demonstrate behavioral, cognitive, and dementia-like impairments [24], and up to 15% of ALS patients are also diagnosed with frontotemporal dementia (FTD) [25,26,27]. The age of onset of the disease in this spectrum varies, and according to the literature, cases under the age of 40 are rare, and for sporadic forms of the disease, the average age is considered to be between 55 and 70 years [28]. Cognitive impairment has been found in up to 70% of patients, with later onset over 65 years resulting in a higher cognitive impairment [29]. Because of this overlap in clinical symptoms, patients are characterized as either ALS-FTD or FTD-ALS [30]. The overlap with the phenotype of the main tauopathies has been studied in terms of specific *MAPT* pathologic phosphorylations of tau, such as the pTau-T175, pTau-T181, and pTau-S396 [31,32], which have been detected in post-mortem examinations of the motor cortex as well as in cerebrospinal tissue. *MAPT* can also influence TDP-43 (the protein product of the *TARDBP* gene) proteinopathies, which can be a direct result of *TARDBP* variants as well as in the form of sporadic TDP-43 proteinopathies [33]. For the sporadic form of the disease, there have been studies that identify genetic variants in the *MAPT* gene as potential risk factors for ALS [34], further emphasizing the complex genetic background of this disorder. While there are no studies that specifically investigate *MAPT* haplotypes affecting the cognitive impairment factor in ALS, there have been observations of the H1 haplotype affecting cognitive impairment through increased expression [35], as well as an increased rate of progression to dementia based on H1 carrier status [36]. While these studies examine the effect of the H1 type in the main group of tauopathies, recent genome-wide association studies (GWAS) have shown that the H1 haplotype is a risk factor for ALS [37] as well, by suggesting that this association is expressed functionally through changes in the speed of axonal transport, which could accordingly lead to a change in the age of onset of the disease and/or the rate of development [21]. The H1 haplotype of the *MAPT* gene has been associated with changes in tau expression, potentially influencing the progression and presentation of ALS comparable to the development of the main group of tauopathies [38]. Available data indicate that changes in tau expression caused by different haplotypes and subtypes could lead to a change in the clinical presentation of classical forms of tau pathologies [38,39,40]. Furthermore, this spectrum of neurodegenerative diseases exhibits an overlap of clinical manifestations that extends to overlap between shared genetic risk loci [37,41,42].

Despite the extensive global research on *MAPT*’s role and function in neurodegenerative disorders, certain geographical and population-specific gaps remain. In Bulgaria, and the world literature, until now, no population studies have been performed that characterize *MAPT* subtypes of the H1 haplotype as risk factors for ALS or ALS-FTD. This study aimed to investigate the significance of these subtypes and the individual variants that compose them in relation to the risk of developing ALS and the age of onset in Bulgarian patients with a sporadic form of the disease.

## 2. Materials and Methods

### 2.1. Patients

The study group consisted of a total of 100 patients (59 male, 41 female) diagnosed with sporadic ALS. The patient group had previously been tested for the most common genetic causes of ALS to verify their sporadic status. Genomic DNA was extracted via desalting methods. Data on the age of onset of the disease and the initial systemic involvement were collected from the Clinic of Neurology, UMBAL “Alexandrovska”, Sofia. Genotypic data for 2504 individuals from the 1000 Genomes Project phase 3 [43] was used as a control group for haplotype estimation and frequencies.

### 2.2. SNP Selection and Genotyping

Six single-nucleotide polymorphisms (SNPs) were selected to characterize the H1/H2 haplotypes and their subtypes (Table 1). The polymorphisms were selected based on previous studies using the same set of polymorphisms to demonstrate the known H1/H2 haplogroup subtypes in the *MAPT* gene [11]. PCR amplification was performed on 6 separate regions of the *MAPT* gene containing the targeted polymorphisms (Table 1).

PCR amplification reactions were carried out in a 25 µL volume containing 50–100 ng of DNA, 0.2 µM of each dNTP, 0.2 µM of each primer, 0.1 U Taq polymerase, and 1x Pol buffer B with 2,5 mM MgCl2. Conditions used for the PCR reaction were as follows: 5 min initial denaturation at 95 °C, followed by 35 cycles at 95 °C for 30 s, 60 °C for 30 s, and 72 °C for 40 s; final extension was conducted at 72 °C for 5 min. Evaluation of the quantity and quality of the obtained amplification products was performed through visualization on agarose gel electrophoresis using a 3% agarose gel. Samples were analyzed in the presence of a molecular marker against which the length of the amplified fragment was read. The products obtained for the variants rs1467967, rs242557, rs1800547, rs3785883, rs2471738, and rs7521 in the *MAPT* gene were sequenced via the direct Sanger sequencing method using the BigDye Terminator v.3.1 sequencing kit (Applera Corporation, Norwalk, CA, USA) and electrophoretic separation on a capillary sequencer (ABI Prism 3130 Sequence Genetic Analyzer, Applied Biosystems, Woburn, MA, USA). The obtained data were automatically processed in the ABI3130 Data Collection Software v3.0 program and obtained in a ready form in the form of an electropherogram.

### 2.3. Statistical Analysis

After determining the allelic frequencies, the results were processed using IBM SPSS Statistics v25 analysis software to perform chi-squared tests and subsequent regression analyses. Mathematical reconstruction of the theoretical haplotypes in the study patient population and the control group was carried out using the expectation–maximization (EM) algorithm implemented in the software Haploview v. 4.2 [44] and a modified version of the algorithm using the Partition–Ligation–Combination/Subdivision (PLC/S) method implemented in the SHEsis Plus software [45].

## 3. Results

Patients were stratified into three age-of-onset groups—32 in the early onset (<50 years), 54 in the standard onset (50–70 years), and 14 in the late onset (>70 years). Allele frequency data were compared to the frequency for the investigated polymorphisms in a global and European control population (Table 2) from the GnomAD project [46].

Chi-squared test and Fisher’s exact test showed that there was no significant difference between the ALS cohort and the GnomAD population frequencies. In the case of the early-onset group, none of the SNPs showed a statistically significant association with age of onset against a threshold of 0.05. These results were replicated for both the standard- and late-onset groups. The same analysis was performed without splitting the patients into groups, which again resulted in no significant associations.

To minimize the population differences, the selected SNPs were tested against the other non-Finnish European population from GnomAd. The results show that rs1467967, rs242557, and rs1800547 have a strong statistically significant association (Table 3) when compared to this population, with rs7521 exhibiting a less robust association. Using data from 961 Bulgarian individuals from GnomAD, we performed a separate test for rs1800547 (REF = 0.7908, ALT = 0.2092), which showed a strong association for this SNP (*p* < 0.00001).

Linear regression analysis was then performed to examine the additive effect of the SNP’s minor allele on the age of onset (Table 4). It is important to note that when conducting this analysis, patients were not divided into groups, and age was examined as an incremental variable. Results showed a statistically significant weak positive correlation between the genetic variant rs1467967 and age of onset (r = 0.182, *p* = 0.035). Conversely, SNPs rs242557, rs1800547, rs3785883, rs2471738, and rs7521 displayed correlations with age of onset, but only rs3785883 and rs2471738 were statistically significant (r = −0.206, *p* = 0.02; r = −0.204, *p* = 0.021, respectively).

**Figure 2:**
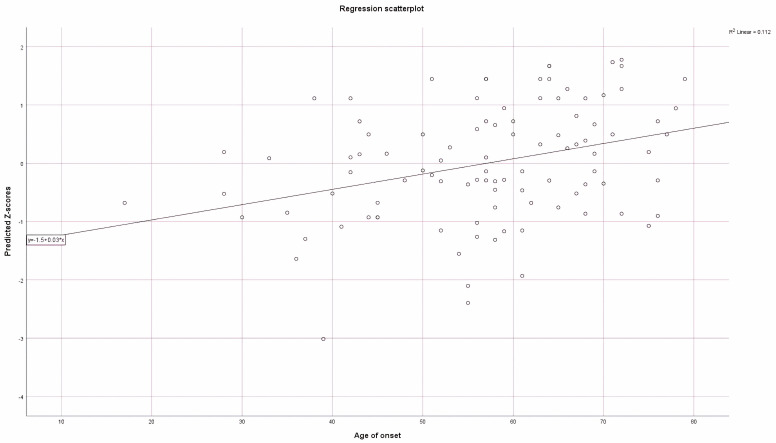
Regression scatterplot for the additive effect of the SNP’s minor allele on the age of onset.

Hierarchical regression using sex as a first predictor followed by the six SNPs showed that neither had statistically significant predictive power on the age of onset alone or in a combined model at the 0.05 level, although rs3785883 is close with a *p*-value of 0.053 (Table 5). The predictive power of the genotypes for the subtype tagging SNPs is weak at R^2^ = 0.112 (Figure 2) and not statistically significant.

Haplotype frequencies, stepwise inheritance analysis, and tests for correlation between haplotype and disease presence were performed using Haploview 4.2 and SHEsisPlus software tools. This was caried out for two models, the first using the previously stratified age-of-onset groups and the second examining all patients as a whole group. In this statistical reconstruction, 200,000 permutations are used to estimate the carrier frequencies of the specific haplotypes more accurately in the samples. The results of the first model are shown in Table 6. None of the haplotypes demonstrated a statistically significant association with the presence of ALS in either group when a threshold of *p* < 0.05 was applied. In an analysis of the correlation between minor allele carrier status and the manifestation of the disease itself, a significant association was found for the single-nucleotide polymorphism rs7521 with the development of ALS (irrespective of age). This association reached a *p*-value of 0.0085; however, this was only found in the standard-age-of-onset group (Table 6).

The second model demonstrated statistically significant results, with a correlation between the specific GGAGCA (H1b) haplotype and disease status after 500,000 permutations. The haplotype has a statistically significant association with ALS against a threshold of *p* < 0.05 (Table 7). Further analysis showed a nearly two-fold increased risk of developing ALS compared to other haplotypes (OR 1.973 and 95% CI 1.279–3.044, against literature data of 0.01% population risk). Compared to control data from 2504 individuals, the haplotype is over-represented in the ALS group (12.5% vs. 6.7%).

**Figure 3:**
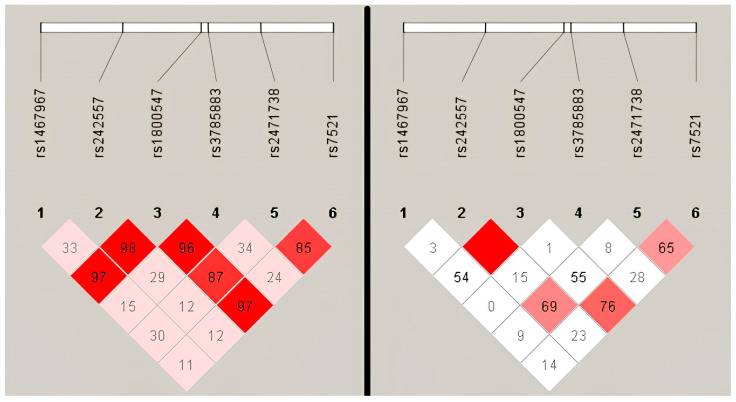
Linkage disequilibrium scores for the control group and the ALS group: white (weak LD)—D’ < 1, LOD < 2; shaded pink/red (stronger LD)—D’ < 1, LOD ≥ 2; bright red (almost complete LD)—D’ = 1, LOD ≥ 2. Numbers in the squares depict D’ values, where D’ = 1.0 when no number is given.

Plotting the linkage disequilibrium (LD) showed that in the patient group, the linkage is not as complete as in the 1000 Genomes group as shown in Figure 3. While in the 1000 Genomes the linkage seems almost complete for rs1800547, the H1 tagging marker, and the rest of the markers, in the patient group we can see that only rs242557 appears to be completely linked to rs1800547.

To further validate the association, the analysis was repeated using the SHEsisPlus software. The H1b haplotype’s association remains statistically significant with disease status following test correction methods (Table 8). Interestingly, the haplotypes H1h, H1r, and H1j have a small negative association with disease status (indicated by their OR), which has minimal statistical significance when using the Benjamini–Hochberg False Discovery Rate correction.

## 4. Discussion

The stratification of the initial patient cohort based on the age of ALS onset provides a comprehensive approach to understanding the genetic underpinnings of the disease across different age groups. The categorization into early-, standard-, and late-onset groups aligns with the broader literature that suggests age-related variations in the genetic risk factors for ALS. The importance of considering other factors such as gender, ethnicity, or geographical location is evident in the literature. For instance, a study on Chinese ALS patients found that they have an earlier age of onset compared to European and Japanese patients and are more likely to have bulbar onset, which is related to rapid progression and shorter survival [47]. Such findings underscore the importance of considering diverse cohorts to capture the full genetic landscape of ALS. This is further emphasized when comparing allele frequency data available from the GnomAD project. We discovered several associations for rs1467967, s242557, and rs1800547, with a weaker association for rs7521 when analyzing the patient cohort against the other non-Finnish European individuals data from GnomAD (Table 3). Furthermore, a strong association was found for the H1 haplotype when using data from Bulgarian individuals from the GnomAD database, which is consistent with previous findings linking the H1 haplotype to ALS [37]. We also observed that rs7521 was associated with disease status in the standard-age-of-onset group. This association was not observed when we applied the second model, suggesting that outliers in terms of age of onset may be influenced by different loci. With regard to the early-onset group, it is likely that that the patients with an early age of onset harbor single (or a handful of) high-impact variants, which would diminish the potential effect of other factors with a lower impact. Nevertheless, these observations align with ALS’s complex genetic nature, where multiple genes and environmental factors may contribute to disease risk.

While reports have associated the parent H1 type with ALS occurrence [21], this is the first study to associate a specific *MAPT* H1 subtype with ALS. Our results show that a specific haplotype—GGAGCA (H1b)—for the *MAPT* gene is over-represented in the Bulgarian ALS population, with a statistically significant association for the risk of developing ALS. The H1b haplotype differs from the reference sequences by a single nucleotide change: guanine to adenine located downstream of exon 9. The rs3785883 A allele has been previously associated with higher CSF tau levels in Alzheimer’s disease (AD) and faster disease progression [48,49], but has also been reported as a protective factor for AD [50]. Interestingly, there are no singular associations for the G allele, part of the H1b haplotype we discovered to confer a risk for ALS. Regarding the haplotypes associated with a lower than 1 odds ratio for the disease (H1h, H1r, and H1j), they share a common difference from the H1b: the A allele of rs1467967. This allele has been associated with an increased level of total tau in a Croatian AD cohort [51]. Although only the H1b haplotype was associated with ALS, the locations of the specific variants may point towards delicate transcription mechanisms occurring before the first exon and immediately after the ninth exon of *MAPT*. Aberrant exon 10 splicing can be a risk factor for and a driver of neurodegenerative disorders [7], and potential downregulation of *MAPT* can have a small protective effect for such disorders. Combined with the observed results, we can speculate that these SNPs have a small yet possibly observable effect on the transcriptional regulation of the gene and its splicing, based on location alone. Future studies could explore this concept in more detail.

Another interesting observation arises from the linkage disequilibrium plots observed using the Haploview software (Figure 3). We can see that in the control population from the 1000 Genomes project, the six variants appear to be almost completely linked, which is supported by data indicating that there is no internal recombination within the region defined by the H1 haplotype [52]. When visualizing the linkage between the H1-subtype variants in the control and in the patient group next to one another, we can see that the markers are not inherited together in the patient group, which would support the different genetic background of the ALS patients compared to the controls. Compounding factors that have not been included in this study cannot be excluded, and, again, the size of the patient cohort limits the impact of these observations. 

The lack of a specific Bulgarian control group is a limiting factor for this study, which did not allow us to investigate for any population-specific stratification or confounders. It is possible that the investigated markers could show singular associations, despite their small effect size, in a separate, Bulgarian control group. Nevertheless, the broader control group that we decided to use has its strengths, as ALS is not a disease limited to a specific ethnicity or a region. While our future studies will include a local Bulgarian control group, we hope to validate the *MAPT* subhaplotype association across diverse populations.

Both Haploview 4.2 and SHEsisPlus provide a powerful toolkit combined with an intuitive interface. They use the Expectation–Maximization (EM) algorithm to estimate haplotype frequencies, which iteratively refines the estimates of haplotype frequencies to compute the maximum-likelihood haplotype distribution [44,45]. The software tools complement each other, as Haploview allows for a visualization of the linkage between the variants being studied, while SHEsisPlus offers more robust statistical correction methods. A further benefit of both software suites is that they require minimal to no programming knowledge.

The findings presented in this study are novel and significantly augment the existing literature. Up until our research, there has been a conspicuous absence of data linking specific haplotypes in the *MAPT* gene with ALS. This knowledge gap may be a critical piece of the puzzle in understanding the genetic underpinnings of this disease. Our results elucidate a potential association between a particular haplotype in the *MAPT* gene and ALS, shedding light on a previously unexplored genetic avenue. If our initial findings are validated through subsequent studies with larger patient populations, the identified haplotype could emerge as a pivotal prognostic factor. Such a development would be monumental in the realm of predictive diagnosis, enabling clinicians to offer timely and targeted interventions to patients at risk. Not only would this enhance patient care, but it could also pave the way for the development of more effective therapeutic strategies tailored to the genetic profiles of ALS patients.

In conclusion, while the genetic landscape of ALS remains complex, our study provides valuable insights into specific genetic variations and haplotypes that might be associated with the disease. The identification of the GGAGCA (H1b) haplotype as a potential risk factor for ALS warrants further investigation in larger cohorts and diverse populations. Understanding the role of such genetic markers can pave the way for personalized therapeutic strategies and early interventions for ALS.

## Figures and Tables

**Table 1:** RS identifiers and genomic location of the H1 subtypes tagging SNPs.

SNP	Location (GRCh37)
rs1467967	Chr. 17:g.45908813G>A
rs242557	Chr. 17:g.45942346G>A
rs1800547	Chr. 17:g.45974480A>G
rs3785883	Chr. 17:g.45977067A>G
rs2471738	Chr. 17:g.45998697C>T
rs7521	Chr. 17:g.46028029A>G

**Table 2:** Allelic frequencies for the tagging SNPs from the GnomAD project and the patient cohort; REF, reference allele; ALT, alternate allele at the genomic position.

			Worldwide GnomAD		European GnomAD		NFEuropean GnomAD		Patients sALS	
SNP	REF	ALT	REF	ALT	REF	ALT	REF	ALT	REF	ALT
rs1467967	G	A	0.348	0.652	0.335	0.665	0.306	0.694	0.415	0.585
rs242557	G	A	0.636	0.364	0.631	0.369	0.659	0.341	0.54	0.46
rs1800547	A	G	0.786	0.214	0.783	0.217	0.781	0.219	0.86	0.14
rs3785883	A	G	0.187	0.813	0.181	0.819	0.173	0.827	0.175	0.825
rs2471738	C	T	0.814	0.186	0.783	0.217	0.795	0.205	0.795	0.205
rs7521	A	G	0.133	0.867	0.46	0.54	0.44	0.56	0.525	0.475

**Table 3:** Results from chi-squared test comparing allele frequencies from the ALS cohort against other non-Finnish European individuals from GnomAD. NFEuropean, other non-Finnish European.

SNP	NFEuropean REF	NFEuropean ALT	*p*-Value	Result
rs1467967	0.306	0.694	**0.001542**	Significant at *p* < 0.01.
rs242557	0.659	0.341	**0.000746**	Significant at *p* < 0.01.
rs1800547	0.781	0.219	**0.007059**	Significant at *p* < 0.01.
rs3785883	0.173	0.827	0.944699	Not significant at *p* < 0.05
rs2471738	0.795	0.205	0.998499	Not significant at *p* < 0.05
rs7521	0.44	0.56	**0.020821**	Not significant at *p* < 0.01, Significant at *p* < 0.05

**Table 4:** Results obtained from the linear regression analysis.

Correlations								
		Onset	rs1467967	rs242557	rs1800547	rs3785883	rs2471738	rs7521
**Pearson Correlation**	Onset	1.000	0.182	−0.136	−0.066	−0.206	−0.204	0.037
	rs1467967	0.182	1.000	−0.026	0.125	−0.001	−0.048	0.114
	rs242557	−0.136	−0.026	1.000	0.289	0.079	0.412	−0.227
	rs1800547	−0.066	0.125	0.289	1.000	−0.009	0.126	0.280
	rs3785883	−0.206	−0.001	0.079	−0.009	1.000	0.091	−0.106
	rs2471738	−0.204	−0.048	0.412	0.126	0.091	1.000	−0.313
	rs7521	0.037	0.114	−0.227	0.280	−0.106	−0.313	1.000
**Sig. (1-tailed)**	Onset	.	0.035	0.089	0.256	0.020	0.021	0.357
	rs1467967	0.035	.	0.398	0.108	0.495	0.317	0.130
	rs242557	0.089	0.398	.	0.002	0.219	0.000	0.012
	rs1800547	0.256	0.108	0.002	.	0.466	0.105	0.002
	rs3785883	0.020	0.495	0.219	0.466	.	0.184	0.147
	rs2471738	0.021	0.317	0.000	0.105	0.184	.	0.001
	rs7521	0.357	0.130	0.012	0.002	0.147	0.001	.

**Table 5:** B values and significance for each of the SNPs from the correlation analysis.

Model	Unstandardized Coefficients		Standardized Coefficients	t	Sig.	95.0% Confidence Interval for B	
	B	Std. Error	β			Lower Bound	Upper Bound
(Constant)	69.006	8.672		7.957	0.000	51.785	86.227
rs1467967	3.106	1.667	0.184	1.864	0.065	−0.203	6.416
rs242557	−0.787	1.937	−0.046	−0.406	0.685	−4.633	3.059
rs1800547	−1.034	2.874	−0.040	−0.360	0.720	−6.741	4.673
rs3785883	−4.713	2.408	−0.193	−1.957	0.053	−9.495	0.069
rs2471738	−3.572	2.319	−0.172	−1.540	0.127	−8.178	1.034
rs7521	−0.979	1.923	−0.057	−0.509	0.612	−4.798	2.840

**Table 6:** Haplotype and individual SNP risk association results from 200,000 permutations inHaploview for the three age groups.

Early Onset (<50 years)			Standard Onset (50–70 years)			Late Onset (>70 years)		
Name	Chi Square	*p*-Value	Name	Chi Square	*p*-Value	Name	Chi Square	*p*-Value
Block 1: AAAACA	9.427	0.1316	rs7521	13.289	**0.0085**	Block 1: AGGGCG	4.783	0.4493
rs3785883	3.954	0.5647	Block 1: AAAGCA	6.449	0.1498	rs1800547	4.726	0.4535
Block 1: AGAGCG	2.606	0.7569	Block 1: GGAGCA	5.325	0.2439	rs2471738	2.374	0.8133
Block 1: AAAGTG	2.504	0.7652	Block 1: AGAACA	5.033	0.2873	rs3785883	2.157	0.8756
rs467967	2.212	0.8576	Block 1: GAAGTG	4.096	0.4229	Block 1: GAAGCA	1.887	0.9037
rs1800547	1.884	0.9197	rs242557	3.645	0.5165	Block 1: AGAGTG	1.653	0.9262
Block 1: GAAGCA	1.581	0.9412	Block 1: AGAGCG	3.625	0.5186	rs7521	1.635	0.9356
Block 1: AGAACA	1.437	0.9511	Block 1: GAAGCG	3.332	0.5759	Block 1: GGAGCA	1.454	0.9526
rs7521	1.309	0.9721	rs1800547	2.806	0.7012	Block 1: AGAGCG	1.316	0.9634
Block 1: GAAGCG	1.246	0.9752	Block 1: AGAGTG	2.673	0.7291	Block 1: AGAGCA	1.059	0.9884
Block 1: AAAGCA	1.226	0.9776	Irs2471738	2.201	0.8312	Block 1: GGAGTG	0.683	0.9988
Block 1: AGAGTG	1.209	0.9784	rs3785883	2.147	0.8462	Block 1: GGAACA	0.679	0.9988
Block 1: GGAGCA	1.186	0.9792	Block 1: GGAGCG	1.843	0.9028	rs1467967	0.57	0.9996

**Table 7:** Results from 500,000 permutations in Haploview for the total patient group.

Name	Chi Square	Permutation *p*-Value
Block 1: GGAGCA	8.287	0.0392
Block 1: AAAGCA	6.951	0.0699
Block 1: AGAGCG	6.452	0.0902
Block 1: AGAGTG	4.944	0.1911
Block 1: GAAGCG	4.139	0.2968
Block 1: AGGGCG	3.328	0.454
Block 1: AGAACA	2.838	0.5685
Block 1: AGAACG	2.742	0.5952
Block 1: GAAGTG	2.402	0.6878
Block 1: AAAGTG	2.092	0.7663
Block 1: AAAACA	1.774	0.8585
Block 1: AGAGCA	1.739	0.866
Block 1: GGAGCG	1.507	0.9131

**Table 8:** Results from the haplotype analysis performed using the SHEsisPlus software. Holm—Holm–Bonferroni; SidakSS—Sidak Stepwise; SidakSD—Sidak Step Down; FDR_BH—False Discovery Rate using Benjamini–Hochberg; FDR_BY—False Discovery Rate using Benjamini–Yekutieli.

Haplotype	Case (freq)	Control (freq)	Fisher’s *p*	Pearson’s *p*	OR [95% CI]	Holm	SidakSS	SidakSD	FDR_BH	FDR_BY
***GGAGCA (H1b)***	***0.125***	***0.067***	***0.004***	***0.001***	***1.973 [1.279~3.044]***	***0.019***	***0.06***	***0.018***	***0.002***	***0.01***
AGGGCG	0.115	0.086	0.161	0.164	1.369 [0.877~2.138]	0.659	0.998	0.513	0.179	0.75
GAAGCG	0.085	0.145	0.013	0.015	0.543 [0.328~0.898]	0.14	0.438	0.132	0.019	0.082
AGAGCA	0.11	0.084	0.199	0.213	1.332 [0.846~2.098]	0.659	0.999	0.513	0.219	0.916
GAAGCA	0.07	0.076	0.891	0.742	0.911 [0.524~1.584]	0.742	1	0.742	0.742	1
AAAGTG	0.085	0.053	0.056	0.051	1.656 [0.992~2.763]	0.306	0.848	0.27	0.059	0.247
*AGAACA (H1h)*	*0.04*	*0.082*	*0.033*	*0.03*	*0.462 [0.226~0.944]*	*0.21*	*0.667*	*0.192*	*0.036*	*0.15*
*AGAGTG (H1r)*	*0.02*	*0.061*	*0.013*	*0.015*	*0.312 [0.115~0.846]*	*0.14*	*0.433*	*0.132*	*0.019*	*0.082*
*AGAGCG (H1j)*	*0.035*	*0.086*	*0.006*	*0.009*	*0.381 [0.178~0.815]*	*0.098*	*0.3*	*0.094*	*0.013*	*0.054*
AAAGCG	0.025	0.043	0.281	0.208	0.566 [0.23~1.389]	0.659	0.999	0.513	0.219	0.916
AGAACG	0.02	0.045	0.113	0.082	0.423 [0.156~1.15]	0.413	0.955	0.35	0.092	0.388

## Data Availability

Genotyping data for the examined patient cohort are available on request. Genotyping data for the 2504 controls from the 1000 Genomes project phase 3 are available through Ensembl (http://www.ensembl.org/, accessed on 25 September 2023).
